# HbpA from *Glaesserella parasuis* induces an inflammatory response in 3D4/21 cells by activating the MAPK and NF-κB signalling pathways and protects mice against *G. parasuis* when used as an immunogen

**DOI:** 10.1186/s13567-024-01344-4

**Published:** 2024-07-29

**Authors:** Zhen Yang, Yiwen Zhang, Qin Zhao, Senyan Du, Xiaobo Huang, Rui Wu, Qigui Yan, Xinfeng Han, Yiping Wen, San-Jie Cao

**Affiliations:** Research Center of Swine Disease, College of Veterinary Medicine, Sichuan Agricultural University, Chengdu, China

**Keywords:** *G. parasuis*, HbpA, proinflammatory cytokines, toll-like receptor, MAPK, NF-κB

## Abstract

*Glaesserella parasuis* is usually a benign swine commensal in the upper respiratory tract, but virulent strains can cause systemic infection characterized by pneumonia, meningitis, and fibrinous polyserositis. The intensive pulmonary inflammatory response following *G. parasuis* infection is the main cause of lung injury and death in pigs. Vaccination has failed to control the disease due to the lack of extended cross-protection. Accumulating evidence indicates that the heme-binding protein A (HbpA) is a potential virulence determinant and a promising antigen candidate for the development of a broader range of vaccines. However, it is not yet known whether HbpA contributes to *G. parasuis* virulence or has any potential immune protective effects against *G. parasuis*. Here, we show that HbpA can induce the transcription and secretion of proinflammatory cytokines (IL-6, TNF-α, and MCP-1) in porcine alveolar macrophages (PAM, 3D4/31). The HbpA protein is recognized by Toll-like receptors 2 and 4 on 3D4/21 macrophages, resulting in the activation of MAP kinase and NF-κB signalling cascades and the transcription and secretion of proinflammatory cytokines. HbpA contributes to virulence and bacterial pulmonary colonization in C57BL/6 mice and plays a role in adhesion to host cells and evasion of the bactericidal effect of pulmonary macrophages. In addition, mice immunized with HbpA were partially protected against challenge by *G. parasuis* SC1401. The results suggest that HbpA plays an important role in the pathogenesis of disease caused by *G. parasuis* and lay a foundation for the development of a subunit or chimeric anti-*G. parasuis* vaccine.

## Introduction

*Glaesserella parasuis*, the etiological agent of Glässer’s disease, is a common inhabitant of the upper respiratory tract of healthy pigs [1]. In stressed or hypoimmune hosts, virulent strains can translocate to the lower respiratory tract, penetrate the mucosal barrier, and enter the bloodstream. The resulting infection causes severe systemic inflammation, characterized by pneumonia, arthritis, meningitis, and fibrinous polyserositis. Glässer’s disease is responsible for heavy economic losses in the pork-producing industry worldwide [2].

The innate immune system is the first line of defence against invading pathogens and relies on pattern recognition receptors to recognize pathogen-associated molecular patterns [3]. Once activated, the system initiates the production of cytokines, chemokines, and other inflammatory factors [4]. Porcine alveolar macrophages (PAMs) offer the first resistance to *G. parasuis* in the lungs. When infected with *G. parasuis*, PAMs recognize the pathogen via toll-like and NOD 1/2 receptors and then regulate the production of a series of cytokines and chemokines through the NF-κB and MAPK signalling pathways [5, 6]. PAMs can clear pathogens by activating a strong inflammatory response [7]. Alternately, macrophages and neutrophils are drawn to the infection site by chemotaxis, resulting in lung tissue damage caused by the excessive production of inflammatory cytokines [8].

To date, 15 serotypes of *G. parasuis* with varying degrees of virulence have been described, but approximately 10% of isolated strains are non-typable [9]. Due to the number of serotypes and the poor cross-protection offered by standard bacterin vaccines, current immunization strategies for controlling Glässer’s disease are inadequate. Additionally, bacterial coinfections such as *G. parasuis* and *Mycoplasma hyorhinis* are common in high-density pig herds [10, 11]. Treatment of Glässer’s disease now relies mostly on antibiotic therapy [2, 12], which is accompanied by a growing risk of antibiotic resistance. For these reasons, there is an urgent need to develop potent broad-acting vaccines against Glässer’s disease.

Antigen immunogenicity is an important consideration in vaccine design. One potentially attractive group of antigens is the various bacterial ATP-binding cassette (ABC) transporters that are responsible for transporting molecules through the cell membrane [13]. These proteins play vital roles in nutrient uptake and virulence and may act as immunogens [14]. Accumulating evidence suggests that they are effective targets for the development of vaccines for numerous bacterial pathogens [15]. Heme-binding protein A (HbpA) is an oligopeptide ABC transporter that was initially identified as a potential component of the heme acquisition pathway in *Haemophilus influenzae* (*H. influenzae*). The transporter is highly conserved among heme-dependent *Haemophilus* species [16, 17]. HbpA is localized to the periplasmic space and transports heme into the cytosol of *H. influenzae* subsequent to the initial binding steps at the cell surface. In addition, HbpA has been identified as a virulence determinant. When HbpA function is eliminated by *hbpA* knockout, the pathogenicity *of H. influenzae* is significantly weakened in mice [18], and heme utilization is decreased. This is also observed in other bacteria, such as *Actinobacillus pleuropneumoniae* [19] and *Mycobacterium tuberculosis* [20]. Zhou et al*.* [21] identified HbpA in the serotype 5 strain SH0165 of *G. parasuis. hbpA* (locus tag: HAPS_0092) is 1596 bp in length and encodes a protein containing 531 amino acids. Zhang et al. [22] reported that HbpA is a differentially expressed virulence-related protein in *G. parasuis*, and Yu et al. [23] demonstrated that HbpA is a potential immunogenic antigen based on the immune response mounted against *G. parasuis* serotype 5 in immunized rabbits.

Virulence factor-related proteins are widely used in subunit vaccine research. However, whether HbpA regulates the virulence of *G. parasuis* and the potential of HbpA as a subunit vaccine candidate against *G. parasuis* have not been reported. Hence, a more thorough understanding of HbpA function during *G. parasuis* infection is necessary. Here, we first aimed to investigate the role of HbpA in the virulence of *G. parasuis*. We measured numerous virulence-related phenotypes, including adhesion ability in NPTr cells, evasion ability in iPAM cells, and the capacity to induce secretion of proinflammatory cytokines via TLR2 and TLR4 and the MAPK and NF-κB signalling pathways. Subsequently, mouse models have been used to evaluate the immunoprotective effects of HbpA protein against *G. parasuis*. Overall, our results lay a foundation for the development of a subunit or chimeric anti-*G. parasuis* vaccine.

## Materials and methods

### Bacterial strains and cell culture conditions

The *G. parasuis* field isolate strain SC1401 [24] (highly virulent, serotype 11) and its derivative SC1401-*ΔhpbA* were cultured in tryptic soy agar/broth (Difco) supplemented with 0.1% (w/v) nicotinamide adenine dinucleotide (Sigma Aldrich) and 5% newborn bovine serum (Solarbio) (TSB++ and TSA++). The *E. coli* expression strain BL21 containing *HbpA*-*His* (rHbpA) was subsequently grown in LB (Invitrogen). 3D4/21 porcine alveolar macrophages were preserved in our laboratory and cultured in RPMI 1640 medium (Gibco, Invitrogen) supplemented with 10% FBS (Gibco, Invitrogen). Newborn porcine tracheal epithelial cells (NPTr) were cultured in DMEM supplemented with 10% FBS. All cell lines were maintained at 37 °C in a humidified 5% CO_2_ atmosphere.

### Preparation of recombinant rHbpA and pET-32a His-tag proteins

*Glaesserella parasuis hbpA* (minus the 63 bp signal sequence) was amplified from SC1401 genomic DNA using primers P1 + P2 (all primers used in this study are listed in Table 1). The PCR products were ligated into pET-28a (+) (HANBIO), generating pET-*hbpA*. The plasmid was transfected into *E. coli* BL21(DE3) cells. Transformed cells were cultured to an optical density at 600 nm (OD600) of 0.5 to 0.6 and then induced with 0.2 mM IPTG for 8 h at 28 °C. HbpA-His (rHbpA) was purified using Ni^+^ affinity chromatography (Bio-Rad). Purified rHbpA was dialyzed in 8 L of PBS for 48 days at 4 °C. The protein was then assayed by SDS-PAGE and Western blotting. The preparation protocol used to purify the pET-32a His-tagged protein was similar to that used for rHbpA. Briefly, pET-32a His-tagged protein was expressed in the *E. coli* strain BL21(DE3). Transformed cells (containing pET-32a) were induced with 0.5 mM IPTG for 8 h at 25 °C for optimum expression. The tagged protein was purified by Ni affinity chromatography (Bio-Rad).

**Table 1:** **Primers used in this study**

Primers	Primer sequences (5′–3′)	Products (bp)
P1	cagcaaatgggtcgc**ggatcc**GCACCGACAAATACATTGGTCA	1575
P2	ctcgagtgcggccgc**aagctt**TTAAGGCTTCAGACTTACGCCAT	
P3	ctatgacatgattacgaattc GGTTACGCTTGGTTGTGT	946
P4	tgtgttttatatttttctcgttcat AATAAGTTCCCTAAGTTGAAAA	
P5	tagctataaattatttaataagtaa TCTATTAGGGAGCTTAAAATATTA	933
P6	caggtcgactctagaggatcc GCGATTGACTAAATAAACATAGT	
P7	ATGAACGAGAAAAATATAAAA	735
P8	TTACTTATTAAATAATTTATAGCTAT	
qPCR primers		
P9(GAPDH)	GTGTTCCTACCCCCAATGTG	189
P10(GAPDH)	CATCGAAGGTGGAAGAGTGG	
P11(IL-6)	GGGACTGATGCTGGTGACAA	147
P12(IL-6)	TCCACGATTTCCCAGAGAACA	
P13(TNF-α)	CGTCAGCCGATTTGCTATCT	184
P14(TNF-α)	CTTGGGCAGATTGACCTCAG	
P15(MCP-1)	AGAAGGAATGGGTCCAGACATA	178
P16(MCP-1)	GTGCTTGAGGTGGTTGTGGA	

### SC1401Δ*hbpA* construction and growth analysis

SC1401Δ*hbpA* was constructed as previously described [25]. Briefly, SC1401Δ*hbpA* was generated via allelic exchange. The 946-bp upstream homologous region and the 933-bp downstream homologous region of the *hbpA* locus were amplified using primers P3 + P4 and P5 + P6, and the 735-bp erythromycin resistance cassette was amplified from plasmid pMG36e using primers P7 + P8. The three fragments were subsequently cloned and inserted into linearized pK18mobsacB to generate pK18-*hbpA*. pK18-*hbpA* was transformed into *G. parasuis* SC1401 using the natural transformation method. An optimized protocol for natural transformation was used as previously described [22]. The growth of the wild-type and SC1401Δ*hbpA* strains was assessed to exclude the possibility of virulence attenuation or enhancement resulting from altered growth characteristics. Briefly, a single colony from the TSA++ culture plate was inoculated into 5 mL of TSB++ culture medium and cultured in a shaker at 37 °C and 220 rpm until the culture reached an optical density at 600 nm (OD_600_) value of 1.0. A 5-µL aliquot of culture was then inoculated into 5 mL of fresh TSB++ culture medium, and growth was monitored by measuring the OD_600_ every hour. All tests were performed in triplicate and repeated three times.

### Detection of proinflammatory cytokines using ELISA and qRT-PCR

ELISA and qRT-PCR were used to determine the ability of HbpA to stimulate cytokine synthesis. 3D4/21 cells were plated into 6-well plates at a density of 1 × 10^6^ cells/well, cultured overnight, and then incubated with 0.2, 2, 20, or 40 μg/mL rHbpA or infected with *G. parasuis* (SC1401 or SC1401Δ*hbpA*) at MOIs of 1 or 10. For the positive control, cells were incubated with 200 ng/mL LPS (Solarbio), and for the negative control, cells were treated with 20 μg/mL pET-32a tag protein (MW: 19 kDa). The cells were incubated for 12 h at 37 °C, and the supernatants and cells were harvested. Cytokine levels in the supernatants were determined by ELISA following the manufacturer’s instructions (Multi Sciences, Hangzhou, China). Briefly, the ELISA plates were soaked in washing solution for 30 s, the wells were emptied, and then 50 μL of supernatant was aliquoted into the test wells. A standard curve was made by aliquoting 100 μL of sample standards into wells. Fifty microliters of diluted biotinylated antibody (anti-MCP-1, anti-IL-6, and anti-TNF-α) was added to all the wells. The plates were incubated for 2 h at room temperature and then washed 6 times with washing buffer. Next, 100 μL of streptavidin-HRP solution (1:100) was added to all the wells, including the blank. The plates were incubated for 30 min at 37 °C and then washed 6 times. Finally, 100 μL of sulfuric acid was aliquoted into each well to stop the enzyme–substrate reaction; the optical density was measured by a full wavelength scanner (Thermo Fisher Scientific, Inc.) at 450 nm. The transcription levels of IL-6, TNF-α, and MCP-1 were measured by qRT-PCR. The primers used for qPCR are listed in Table 1. Total RNA was isolated from cells by using a Total RNA Isolation Kit (Sangon Biotech, Shanghai, China), and complementary DNA (cDNA) was generated from 1 μg of total RNA using HiScript III RT SuperMix (Vazyme, Nanjing, China). To measure RNA expression, a 20 μL mixture containing diluted cDNA was assayed using ChamQ SYBR Color qPCR Master Mix (Vazyme, China) on a LightCycler 480 II (Roche, Switzerland). GAPDH expression was used as an internal control. These experiments were performed three times; in each experiment, there were three technical replicates per condition.

### Western blot

HbpA-induced phosphorylation of p38, Erk1/2, JNK, and p65 (key proteins in the MAPK/NF-κB signalling pathway) and activation of TLR2 and TLR4 were analysed by Western blotting. 3D4/21 cells were seeded in a 6-well plate at a density of 1 × 10^6^ cells/well and cultured overnight, after which 20 μg/mL rHbpA or pET-32a tag protein was added, followed by incubation for 12 h. Supernatants and cell lysates were harvested and treated as described previously [26]. Briefly, the collected cells were suspended in cold RIPA lysis buffer supplemented with protease inhibitors, serine/threonine phosphatase inhibitors, and tyrosine protein phosphatase inhibitors and incubated in an ice bath for 15 min. Fifty micrograms of protein from each sample was subjected to 12.5% SDS-PAGE and transferred to PVDF membranes (Millipore). The membranes were blocked with 5% skim milk for 1.5 h at 25 °C and then incubated with primary antibodies overnight at 4 °C. The primary antibodies used are listed in Table 2. The membranes were washed again and incubated with HRP-linked goat anti-rabbit antibodies (1:5000) for 2 h at 25 °C. The bands were developed using ECL according to the manufacturer’s protocol. Bands were detected by the ChemiDocTM XRS+ system with Image LabTM software (Bio-Rad).

**Table 2:** **The details of primary antibodies used in the study**

Antibody name	Dilution ratio	Source	Catalogue number	Molecular weight (kDa)
p38-MAPK	1:1000	Rabbit	Abcam ab31828	41
p-p38-MAPK	1:500	Rabbit	Abcam ab47363	41
Erk1/2	1:2000	Rabbit	Abcam ab184699	42/44
p-Erk1/2	1:2000	Rabbit	Abcam ab278538	42/44
JNK	1:1000	Rabbit	Abcam ab179461	48
p-JNK	1:1000	Rabbit	Abcam ab76572	48
p65	1:2000	Rabbit	Abcam ab16502	60
p- p65	1:5000	Rabbit	Abcam ab86299	60
TLR2	1:500	Rabbit	Abcam ab213676	89
TLR4	1:500	Rabbit	Abcam ab13556	89

### Effects of MAPK and NF-κB inhibitors

To assess the roles of the p38, ERK1/2, JNK-MAPK, and NF-κB signalling pathways in the rHbpA-mediated production of cytokines, specific inhibitors against each pathway were used. We treated rHbpA-stimulated macrophages with an Erk1/2 inhibitor (U0126), a JNK inhibitor (SP600125), and an NF-κB inhibitor (PDTC) and then assayed the secreted IL-6, TNF-α, and MCP-1. The concentration of inhibitors in all cases was 10 μM, as in our previous study [26]. 3D4/21 cells were plated into 6-well plates at a density of 1 × 10^6^ cells/well, cultured overnight, and then incubated with 10 μM SB203580 (p38 inhibitor), SP600125 (JNK inhibitor), U0126 (ERK1/2 inhibitor), or PDTC (NF-κB inhibitor) for 2 h at 37 °C prior to incubation with 20 μg/mL rHbpA for 12 h. Inhibitors for studying signalling pathways were obtained from MCE (10 mM each, Monmouth Junction, NJ, USA) and dissolved in DMSO. An equal volume of DMSO was added to the cells for the control. Culture supernatants were collected, and the levels of IL-6, TNF-α, and MCP-1 were determined by ELISA using a commercial kit (Multi Sciences).

### TLR blocking assay

To determine the pattern recognition receptors for HbpA on 3D4/21 macrophages, we blocked toll-like receptors 2 (TLR2) and 4 (TLR4) using Ultra-LEAF™-purified CD282 (TLR2) (BioLegend, Cat: 153,001) and LEAF™-purified anti-mouse TLR4 (CD284)/MD2 (BioLegend, Cat: 117617). Each antibody was used at a final concentration of 8 µg/mL. 3D4/21 cells were plated into 6-well plates at a density of 1 × 10^6^ cells/well, cultured overnight, incubated for 2 h with a single antibody, and then incubated overnight with 20 μg/mL rHbpA protein. Cell culture supernatants were collected for detection of secreted cytokines using ELISA kits as described in “Detection of proinflammatory cytokines using ELISA and qRT-PCR” section.

### Adhesion and macrophage phagocytosis

Adhesion and macrophage phagocytosis assays were used to investigate the possible reduction in the infection capacity of SC1401Δ*hbpA*. Assays were performed as previously described [27]. For the adhesion assay, newborn porcine tracheal epithelial cells (NPTr) were aliquoted into 6-well plates and incubated at 37 °C until they reached confluence (approximately 1 × 10^6^ cells/well). SC1401 or SC1401Δ*hbpA* cells grown in TSB++ overnight were collected by centrifugation (3000 × *g* for 10 min) and resuspended in DMEM. Cells were infected with SC1401 or SC1401Δ*hbpA* at multiplicities of infection (MOIs) of 1:10 and 1:100 and then incubated for 2 h at 37 °C. The cells were washed six times with sterile PBS to eliminate nonadherent bacteria. Adherent bacteria were quantified by spreading cells on TSA++ plates, incubating for 36 h at 37 °C, and then counting the number of colonies (CFU). The 3D4/21 cells used for the phagocytosis assay were subjected to identical procedures, except that the extracellular bacteria were killed by incubating the cell monolayer with RPMI 1640 containing 25 µg/mL chloromycetin for 1 h.

### Animal challenge

Specific pathogen-free (SPF) C57BL/6 mice were utilized for in vivo experiments. All experimental protocols were approved by the Animal Ethics Committee of Sichuan Agricultural University and were carried out accordingly. All experiments in this study were approved by the Institutional Animal Care and Use Committee of Sichuan Agricultural University (SYXK2019-187). To evaluate the virulence of SC1401 and Δ*hbpA*, thirty-nine 4-week-old female mice were allocated randomly to the SC1401, Δ*hbpA*, and PBS groups, each containing thirteen mice. All mice were intraperitoneally injected with 10^8^ CFU/mouse (an absolute lethal dose). Virulence was evaluated daily for 72 h by careful monitoring of the mice. Three surviving mice were randomly selected for euthanasia 24 h post-infection, and lung tissues were collected for immunohistochemistry (IHC) assays to determine the extent of *G. parasuis* (wild-type strain or *hbpA* deletion mutant strain) colonization in the lungs. IHC was performed according to previous methods [28]. Briefly, harvested lung tissues were fixed in 10% neutral buffered formalin at room temperature overnight and subsequently embedded in paraffin. Paraffin-embedded tissues were cut into 4 μm sections, and 3% H_2_O_2_-methanol was applied to the sections to suppress endogenous peroxidase activity after dewaxing and hydration. IHC to detect the *G. parasuis* wild-type or Δ*hbpA* strains was optimized using an anti-PotD antibody (1:200 dilution) as the primary antibody, which was prepared in our laboratory [29]. IHC-stained slides were observed using an Olympus optical microscope (magnification: 400×). Positive IHC staining corresponded to yellow‒brown colouration, according to the manufacturer’s instructions. IHC staining of *G. parasuis* was quantified using the mean optical density (MOD = IOD SUM/area).

### Immunization and challenge

To investigate the ability of rHbpA to protect against *G. parasuis infection* in vivo, we used forty 4-week-old female mice randomly divided into 4 groups (mock: unimmunized and unchallenged; PBS: immunized with PBS + challenge; adjuvant: immunized with adjuvant + challenge; rHbpA: 150 μg of rHbpA emulsified with adjuvant + challenge). The adjuvant was MONTANIDE™ GEL 01. All the mice were injected subcutaneously with 200 µL. Two booster injections were administered: the first 14 days after the initial immunization and the second 28 days after the initial injection. Seven days after the second booster, all mice (except those in the mock group) were intraperitoneally challenged with 10^8^ CFU (0.2 mL) of the field isolate *G. parasuis* strain SC1401. At 72 h post-challenge, the surviving mice in all groups were humanely sacrificed, and lung tissues were collected to determine the extent of *G. parasuis* SC1401 colonization. Tissues were fixed in neutral-buffered formalin and embedded in paraffin for IHC investigation.

### Statistical analysis

ImageJ (version 1.8.0) was used to quantify the grayscale values of the Western blots. IHC images were captured with CaseViewer and evaluated using ImageJ software. The results of the study were analysed and graphed using GraphPad Prism 8, and the data are expressed as the mean ± standard deviation. Differences between two groups were analysed using Student’s *t* tests. The data from more than two groups were analysed by two-way ANOVA. Significant differences between groups are indicated using asterisks: *p < 0.05, **p < 0.01, ***p < 0.001, and ****p < 0.0001; ns indicates not significant.

## Results

### Preparation of biological materials

**Figure 1:**
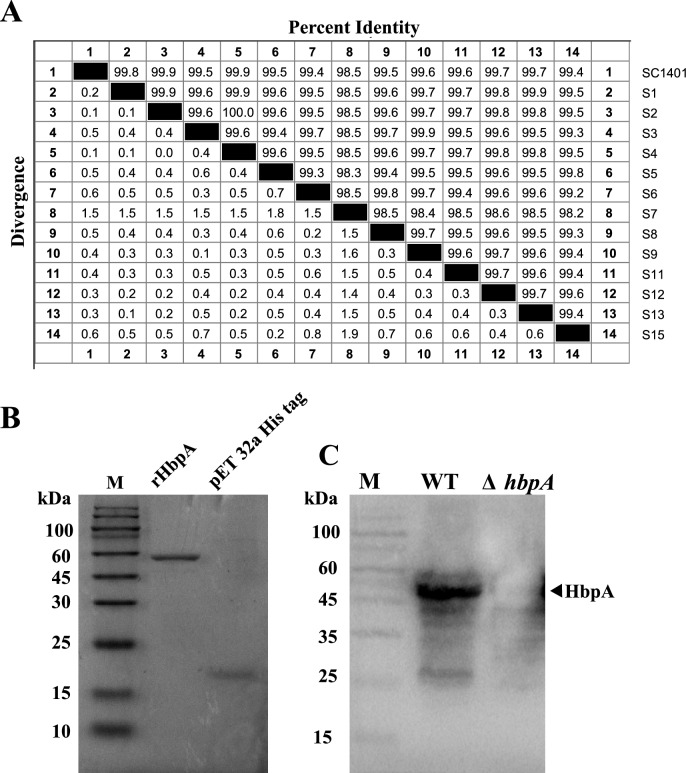
**Expression of recombinant proteins and construction of SC1401Δ*hbpA*.** **A** Sequence identity analysis between different serotype strains. The strain codes used in the present study were as follows: SC1401: field isolate strain Serovar 11; S1 (Serovar 1): 12939, field isolate strain Serovar 1; S2 (Serovar 2): SW140, reference strain; S3 (Serovar 3): SW114, reference strain; S4 (Serovar 4): HPS-1, field isolate strain; S5 (Serovar 5): Nagasaki, reference strain; S6 (Serovar 6):131, reference strain; S7 (Serovar 7):174, reference strain; S8 (Serovar 8): field isolate strain; S9 (Serovar 9):D74, reference strain; S11 (Serovar 11):H465, reference strain; S12 (Serovar 12):ZJ0906, field isolate strain; S13 (Serovar 13):IA-84-17975, reference strain; and S15 (Serovar 15):SD-84-15995, reference strain. There was no genome sequence for serovars 10 and 14. **B** SDS-PAGE of purified HbpA and pET-32a His-tagged protein M: Protein marker. **C** Western blot showing hbpA-deficient *G. parasuis*.

Sequence analysis revealed that HbpA is highly conserved among different serotype strains of *G. parasuis*, indicating that this protein has the potential to serve as a candidate subunit vaccine against this species (Figure 1A). rHbpA and pET-32a tag proteins were expressed with an N-terminal 6×His-tag and purified by Ni-affinity chromatography. The pET-32a His-tag was a recombinant consisting of the 109 amino acids Trx tag thioredoxin protein fused to a 6-histidine tag and was used as the negative control to exclude the possibility of LPS contamination in our study. SDS-PAGE revealed two bands with molecular masses of approximately 58 kDa and 29 kDa, corresponding to the predicted masses of HbpA or thioredoxin protein plus the His tag (Figure 1B). Western blot was performed to detect proteins extracted from the *G. parasuis* Δ*hbpA* and WT strains; HbpA was not detected in SC1401Δ*hbpA*, demonstrating the successful construction of *G. parasuis* Δ*hbpA.* Compared with SC1401, SC1401Δ*hbpA* displayed subtle growth defects, but the difference in their in vitro growth curves was not significant (data not shown).

### rHbpA stimulates the production of IL-6, TNF-α, and MCP-1

**Figure 2:**
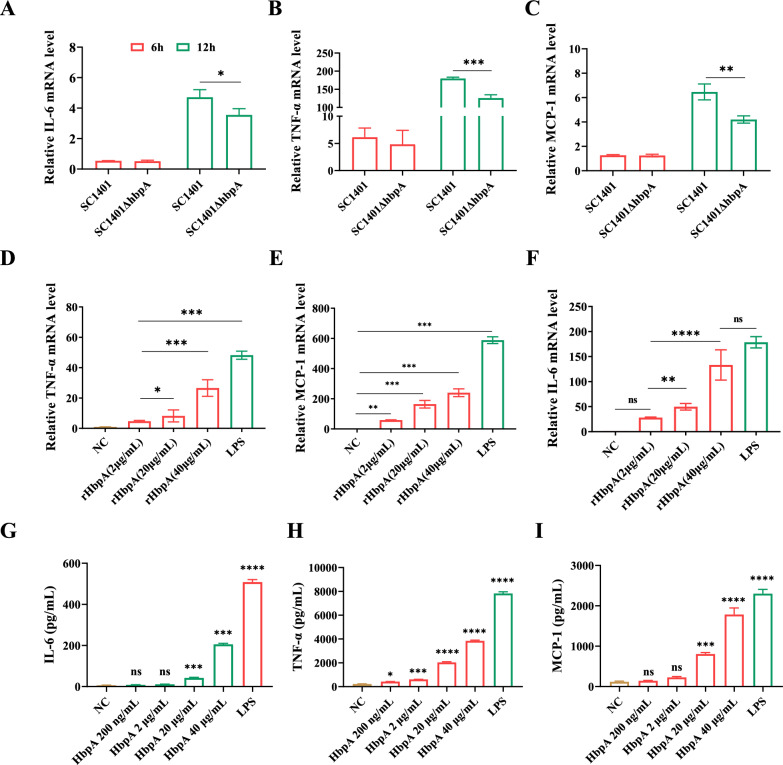
**HbpA induces the secretion of IL-6, TNF-α, and MCP-1.** **A**–**C** 3D4/21 macrophages were incubated with SC1401 or SC1401Δ*hbpA* at an MOI of 10. The bar graphs show the relative IL-6, TNF-α, and MCP-1 mRNA levels. **D**–**F** 3D4/21 macrophages were incubated with 2, 20, or 40 µg/mL rHbpA protein; 100 ng/mL LPS was used as the positive control, and 20 µg/mL pET-32a His-tag protein was used as the negative control. The bar graphs show the relative IL-6, TNF-α, and MCP-1 mRNA levels. The fold change in expression on the y-axis was calculated as the experimental group/negative control. **G**–**I** 3D4/21 macrophages were incubated with 0.2, 2, 20, or 40 µg/mL rHbpA protein; 100 ng/mL LPS was used as the positive control, and 20 µg/mL pET-32a His-tag protein was used as the negative control. The bar graphs show the secreted IL-6, TNF-α, and MCP-1 levels as determined by ELISA. All assays were performed three times.

qRT-PCR revealed that infection with the wild-type (WT) and Δ*hbpA* strains resulted in increased transcription of proinflammatory cytokines in 3D4/21 cells at 12 h post-infection, although the response to SC1401Δ*hbpA* was significantly less than the response to the wild type (Figures 2A–C). Compared to treatment with the pET-32a His-tag protein, treatment with rHbpA resulted in a dose-dependent increase in cytokine transcription (Figures 2D–F). Figures 2G–I show the levels of secreted cytokines stimulated by rHbpA, as determined by ELISA; the increasing trend in secretion was consistent with that observed for transcription.

### rHbpA induces the expression of IL-6, TNF-α, and MCP-1 via the MAPK and NF-κB signalling pathways

**Figure 3:**
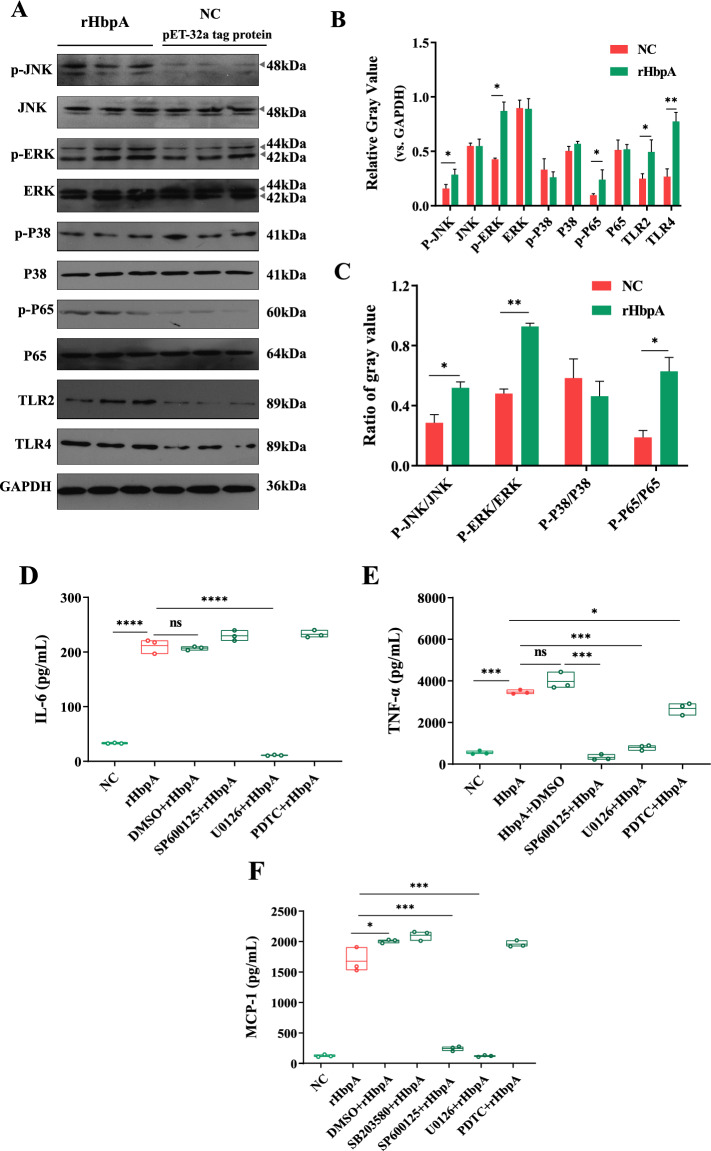
**Identification of the signalling molecules involved in proinflammatory cytokine secretion.** **A**–**C** 3D4/21 macrophages were incubated with 20 µg/mL HbpA protein or pET-32a tag protein for 12 h. **A** Western blot of total protein. **B** Gray values. **C** Ratios of phosphorylated signal molecules to total signal molecules. **D**–**F** Effect of inhibitors on cytokine secretion induced by HbpA. 3D4/21 macrophages were treated with 10 μM U0126 (Erk1/2 inhibitor), 10 μM SP600125 (JNK inhibitor), or 10 μM PDTC (NF-κB inhibitor) and then incubated with 20 µg/mL HbpA protein or His-tag protein for 12 h. The secretion levels of **D** IL-6, **E** TNF-α, and **F** MCP-1 in the culture supernatants were determined by ELISA.

Western blot assays revealed that macrophages incubated with 20 μg/mL rHbpA for 12 h had significantly increased levels of phosphorylated JNK, ERK1/2 and p65 (a signalling molecule in the NF-κB pathway) compared with those in the negative control group (*p* < 0.05) (Figures 3A–C). As shown in Figures 3D–F, Erk1/2 pathway inhibition resulted in significantly lower secretion of each cytokine tested than that in rHbpA-stimulated/unblocked macrophages. JNK pathway inhibition resulted in decreased secretion of TNF-α and MCP-1, and NF-κB pathway inhibition resulted in significantly decreased secretion of TNF-α.

### TLR2 and TLR4 receptors from 3D4/21 cells recognize HbpA

**Figure 4:**
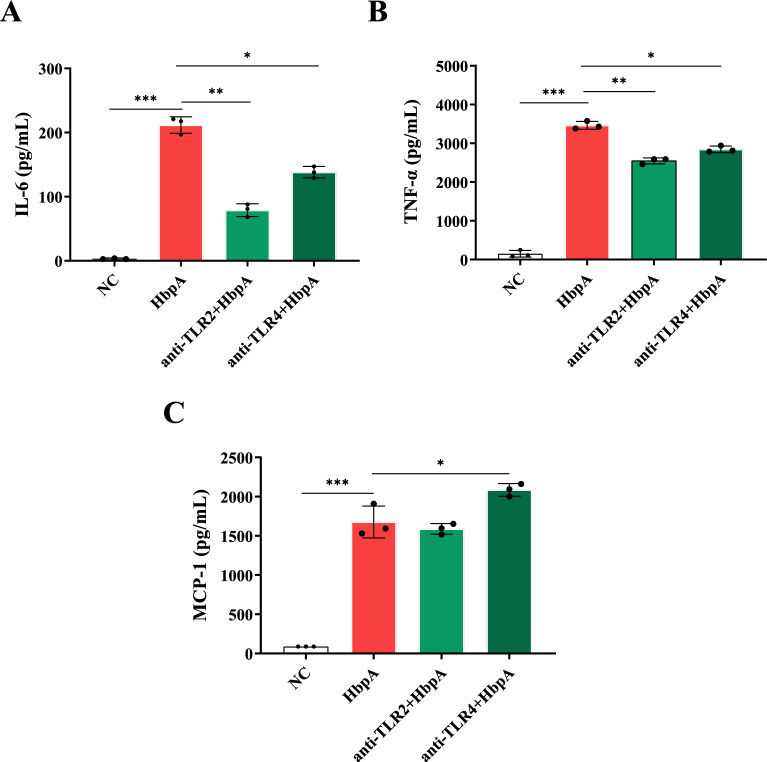
**Analysis of the pattern recognition receptors of HbpA.** A total of 1 × 10^6^ 3D4/21 cells were incubated with 10 µg/mL anti-TLR2 or anti-TLR4 monoclonal antibodies for 2 h and then incubated with HbpA for 12 h. The levels of secreted **A** IL-6, **B** TNF-α, and **C** MCP-1 in the culture supernatants were determined by ELISA.

The TLR2 and TLR4 levels were significantly greater in the HbpA-treated macrophages than in the negative control macrophages (*p* < 0.05) (Figures 3A and B). As shown in Figures 4A–C, the secreted levels of IL-6 and TNF-α were significantly lower in TLR2-blocked cells (*p* < 0.01) than in unblocked cells. The secreted levels of IL-6 and TNF-α were also significantly lower in TLR4-blocked cells (*p* < 0.05).

### HbpA affects bacterial adhesion and host cell survival

**Figure 5:**
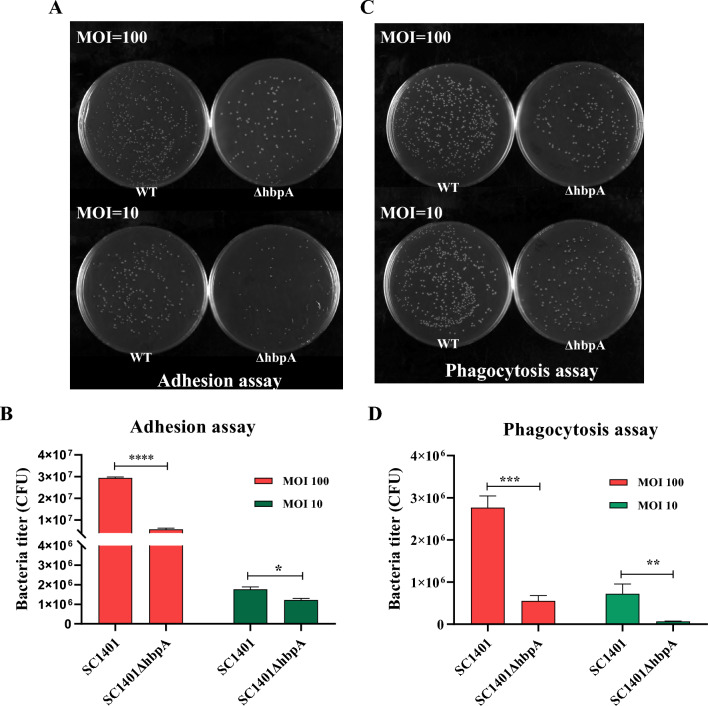
**Adherence of *G. parasuis* to NPTr and viability of phagocytosed bacteria.** **A**, **B** Adherence of SC1401 and SC1401Δ*hbpA to NPTr cells.* The data display the average number of adherent bacteria in each well of a 6-well plate. **C**, **D** Survival of bacteria after phagocytosis by 3D4/21 macrophages. The data display the average number of bacteria in each well of a 6-well plate. Error bars represent the standard errors from three independent experiments.

With respect to NPTr cells incubated with *G. parasuis* (at MOIs of 10 and 100 for 2 h), we found significantly less SC1401Δ*hbpA* attached than to the WT strain (Figures 5A and B). Additionally, for 3D4/21 macrophages incubated with *G. parasuis* (MOI of 10 or 100 for 2 h), significantly fewer SC1401Δ*hbpA*-expressing cells than WT cells survived after phagocytosis by macrophages (Figures 5C and D).

### *ΔhbpA* displays attenuated virulence in vivo

**Figure 6:**
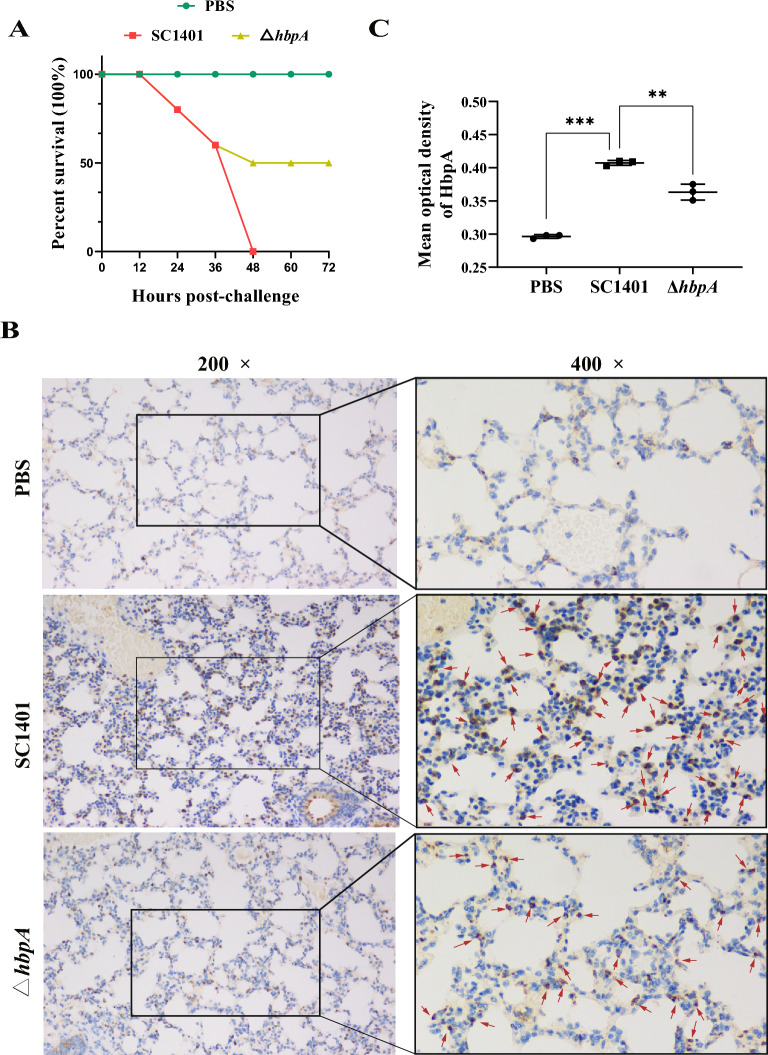
**Survival of mice and bacterial colonization after challenge with a lethal dose (1 × 10**^**9**^** CFU/mouse) of *G. parasuis.*** Images are shown at ×200 and ×400. In each treatment group, ten C57BL/6 mice were challenged with a lethal dose (10^8^ CFU/mouse) of *G. parasuis*. **A** At 48 hpc, all the mice in the SC1401-infected group died, and 50% of the mice in the Δ*hbpA*-infected group died. **B** *G. parasuis* immunohistochemistry showed that SC1401 colonized the lungs of C57BL/6 mice at a relatively high density. Brown dots represent *G. parasuis* or its deletion derivative. **C** The Mean optical density of HbpA was analyzed according to the IHC assays.

Mice were injected with 10^8^ CFU/mouse (0.2 mL) of wild-type or Δ*hbpA* SC1401 and observed for 72 h. Injection with WT resulted in 100% lethality within 48 h (10/10); in contrast, injection with Δ*hbpA* resulted in only 50% lethality (5/10) over 72 h (Figure 6A). Animal challenge experiments were also conducted to verify colonization by *G. parasuis* strains. IHC was used to detect colonized *G. parasuis* in the lung. The IHC results demonstrated that wild-type strains colonized at significantly greater levels than did the *hbpA* deletion strains (Figures 6B and C).

### rHbpA protects against *G. parasuis* in challenged mice

**Figure 7:**
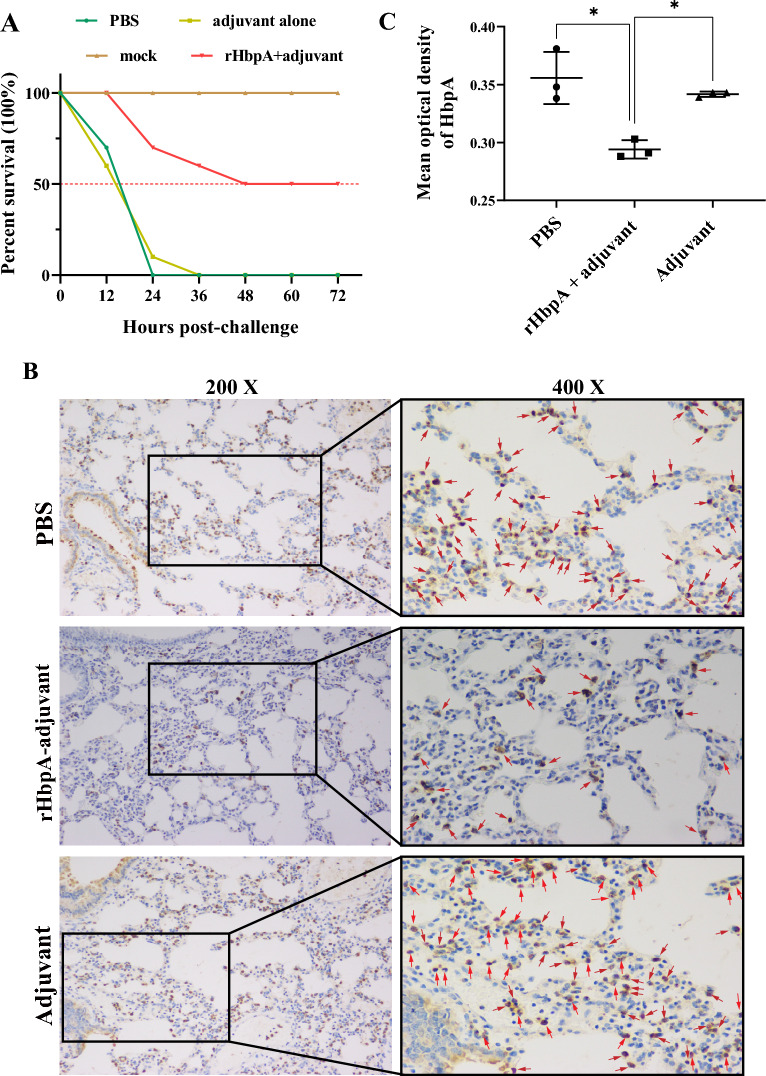
**rHbpA protects mice from *G. parasuis*.** In each experimental group, ten C57BL/6 mice were challenged with a lethal dose (10^8^ CFU/mouse) of *G. parasuis* SC1401. **A** At 24 hpc, all the mice in the PBS group died, 90% of the mice in the adjuvant alone group died, and 20% of the mice in the rHbpA+ adjuvant group died. At 48 hpc, 50% of the mice in the rHbpA+ adjuvant group died, and the surviving mice survived until they were euthanized at 72 hpc. **B** *G. parasuis* immunohistochemistry showed that SC1401 colonized at a greater density in the mock and adjuvant groups. Brown dots represent *G. parasuis*. Lung tissues were collected at 24 hpc and prepared for the IHC assay. **C **The Mean optical density of HbpA was analyzed according to the IHC assays.

We tested the protective effect of rHbpA vaccination against *G. parasuis* SC1401 challenge in mice. As shown in Figure 7A, 100% of the mice in the PBS group and 90% of the mice in the adjuvant alone group died 24 h after challenge. In contrast, in the rHbpA+ adjuvant group, 20% of the mice died after 24 h, and 50% of the mice died after 48 h (hereafter, there was no further mortality). IHC was used to detect colonized *G. parasuis* in the lungs of the mice in each group. The IHC results demonstrated that SC1401 in the mock and adjuvant groups colonized at significantly greater levels than in the rHbpA-adjuvant group, indicating an increase in the bacterial clearance capacity of the rHbpA-vaccinated mice (Figures 7B and C).

## Discussion

*Glaesserella parasuis* is a common inhabitant of the upper respiratory tract in swine, but under certain circumstances, it can cause a systemic infection called Glässer’s disease, characterized by fibrinous polyserositis and pneumonia. At present, disease control depends mainly on herd management, followed by the use of antimicrobial agents and immunization via vaccination. Many pig farms have management problems, such as early weaning, transportation of weaned pigs, inappropriate indoor temperature and humidity, or infection of animals with pathogens that can trigger the onset of Glässer’s disease if virulent *G. parasuis* is present [30]. Antimicrobials are widely used for treating Glässer’s disease, resulting in drug and multidrug resistance in *G. parasuis* isolates [31]. Additionally, antimicrobial agents can prevent the development of an effective protective immune response against future infections caused by virulent *G. parasuis*. The use of antimicrobials around the time of colonization of the upper respiratory tract can interfere with the colonization by *G. parasuis*, and importantly with the onset of immunity and posterior protection of the piglets [32]. Vaccination is another strategy to reduce the potential increase in Glässer’s disease caused by antibiotic-resistant *G. parasuis*. Vaccination with sole-serotype inactivated bacterins has eased the losses associated with Glässer’s disease, but because of serovar type heterogeneity, these vaccines do not produce broad immunity against the disease [33]. There is, in fact, a significant difference in protection against homologous serovars such as 4 and 5 (the most frequently identified serovars) [34]. Subunit vaccines that contain specific antigenic molecules of *G. parasuis* have the potential to solve this problem. To date, multiple recombinant proteins from *G. parasuis* have been identified as highly immunoreactive, but there are no subunit vaccines that provide effective protection [10]. In light of this, the identification of conserved surface antigenic proteins has become a focus in the development of more effective vaccines, particularly ABC transporters, which are found exclusively in prokaryotes [35]. Moreover, numerous virulence factors, such as the outer membrane protein OmpP2, have been reported to have an immune-protective effect against *G. parasuis* [36]. OmpP2 can induce cells to secrete proinflammatory cytokines [37]; it is a dominant antigen that can rapidly induce the production of high-titre, specific antibodies. HbpA, an ABC transporter, is also highly conserved in *G. parasuis*, so we evaluated its role in virulence and its effectiveness as an immune-protective antigen.

Huang et al. reported that excessive and persistent production of proinflammatory cytokines was responsible for severe pulmonary injury sustained by the host [38]. To investigate the effect of HbpA on the inflammatory response in PAMs, IL-6, TNFα, and MCP-1 transcript levels were quantified in cells induced by SC1401, SC1401*hbpA*, and rHbpA. The results showed that HbpA of *G. parasuis* induces the transcription and secretion of proinflammatory cytokines, including IL-6, TNF-α, and MCP-1, in PAM cells. Western blot analysis and TLR blocking assays demonstrated that HbpA is a ligand of TLR2 and TLR4 in 3D4/21 cells. Our results are consistent with a previous study of the YfeA protein in *G. parasuis,* whose receptors are TLR4 and TLR2 [26]. However, a report describing the PotD protein in *G. parasuis*, whose receptor is TLR4 [29], suggested that the proinflammatory response in 3D4/21 cells is specific for the HbpA protein and not only due to a general proinflammatory response produced by macrophages towards any bacterial protein/LPS. We found that after HbpA was recognized by TLR2 and TLR4, the ERK1/2, JNK-MAPK, and NF-κB signalling pathways were activated to prime proinflammatory cytokine production. Previous studies have shown that *G. parasuis* induces the activation of the NF-κB and MAP kinase signalling pathways via Toll-like receptors [5].

In this study, we found that HbpA-deficient *G. parasuis* (Δ*hbpA*) was significantly less adherent to NPTr cells than was the wild-type pathogen. The adherence of bacteria to host cell surfaces is an essential determinant of bacterial pathogenicity. Adhesion is the initial step in colonization and cellular invasion, leading to persistence in the host, invasion into deeper tissues, and ultimately systemic disease [39]. Although HbpA is not involved in resistance to phagocytosis by PAMs, we found that *G. parasuis* Δ*hbpA* was significantly less viable than the WT strain after phagocytosis by 3D4/21. PAM is a vital line of defence against *G. parasuis* infection [40]. Once bacteria are phagocytosed by alveolar macrophages and internalized to mature phagolysosomes, they are exposed to damaging reactive oxygen species, reactive nitrogen species, low pH, and an array of hydrolytic enzymes [41]. Our results suggest that HbpA plays a role in evading the bactericidal effect of pulmonary macrophages.

Animal infection experiments were performed to assess the contribution of the HbpA protein to bacterial virulence in vivo. The results showed that mortality was greater and faster in WT-infected mice than in Δ*hbpA*-infected mice. IHC showed that wild-type *G. parasuis* colonized the lungs of mice to a statistically greater extent than did Δ*hbpA*. In addition, the results of bacterial phagocytosis and mouse challenge assays showed that fewer Δ*hbpA* bacteria were recovered. These results indicate that fewer bacteria interact with 3D4/21 cells, which might indirectly decrease the induction of cytokines.

Numerous studies have shown that mice, including C57BL6 and BALB/c mice, are a relevant animal model for studying *G. parasuis* pathogenesis. Prior to testing the immune efficacy of the HbpA vaccine in pigs, we tested its protective effect in *G. parasuis-*challenged BALB/c mice. The survival rate of mice immunized with the rHbpA+ adjuvant against *G. parasuis* infection was 50%, whereas that of unimmunized mice was 0%. Additionally, the lungs of unimunized mice contained significantly more *G. parasuis* than did those of rHbpA+ adjuvant-immunized mice, indicating that the ability of rHbpA+ adjuvant-immunized mice to clear bacteria significantly increased. These results demonstrated that rHbpA+ adjuvant vaccination protects mice against a lethal challenge of *G. parasuis*. Because some recombinant proteins that have displayed protection in mice have failed to do so in pigs [42], further investigation on the efficacy of HbpA vaccination in pigs is needed. As adjuvant selection impacts the efficacy of immunogens (38), the 50% survival rate in mice treated with rHbpA+ adjuvant (MONTANIDE™ GEL 01) may be improved with a different adjuvant. An improvement in survival may also be achieved by vaccines containing multiple antigens, including HbpA. In addition, a vaccine that provides protection against *G. parasuis* and some porcine respiratory viruses and/or bacteria may be a research project in the future.

In summary, we have demonstrated that the HbpA protein is a potential virulence factor in *G. parasuis*: it is recognized by TLR2 and TLR4, promotes the secretion of proinflammatory cytokines via the ERK1/2, JNK-MAPK, and NF-κB signalling pathways, and contributes to survival in vitro and in vivo. Moreover, immunization with HbpA provides *G. parasuis*-challenged mice with moderate protection (50% protection) against the pathogen.

## Data Availability

All data underlying the results are available as the article and no additional source data is required.
